# Comparative Study on the Removal Efficiency of Clomazone and Amitriptyline via Adsorption and Photocatalysis in Aqueous Media: Kinetic Models and Toxicity Assessment

**DOI:** 10.3390/ma17061369

**Published:** 2024-03-16

**Authors:** Nataša Tot, Vesna Despotović, Sanja Panić, Branko Kordić, Nina Finčur, Jovana Prekodravac, Dimitar Jakimov, Predrag Putnik, Biljana Abramović, Daniela Šojić Merkulov

**Affiliations:** 1 Technical College of Applied Sciences in Zrenjanin, Đorđa Stratimirovića 23, 23000 Zrenjanin, Serbia; natasazec993n@gmail.com; 2 Department of Chemistry, Biochemistry and Environmental Protection, University of Novi Sad Faculty of Sciences, Trg Dositeja Obradovića 3, 21000 Novi Sad, Serbia; branko.kordic@dh.uns.ac.rs (B.K.); nina.fincur@dh.uns.ac.rs (N.F.); biljana.abramovic@dh.uns.ac.rs (B.A.); daniela.sojic@dh.uns.ac.rs (D.Š.M.); 3 Faculty of Technology, University of Novi Sad, Bulevar cara Lazara 1, 21000 Novi Sad, Serbia; sanjar@tf.uns.ac.rs; 4 Department of Laboratory for Radiation Chemistry and Physics “Gamma” 030, Vinča Institute of Nuclear Sciences, National Institute of the Republic of Serbia, University of Belgrade, 11080 Belgrade, Serbia; prekodravac@vin.bg.ac.rs; 5 Oncology Institute of Vojvodina, Faculty of Medicine, University of Novi Sad, Put Doktora Goldmana 4, 21204 Sremska Kamenica, Serbia; dimitar.jakimov@mf.uns.ac.rs; 6 Department of Food Technology, University North, Trg dr. Žarka Dolinara 1, 48000 Koprivnica, Croatia

**Keywords:** clomazone, amitriptyline, carbon nanotubes, TiO_2_, kinetics, toxicity assessment

## Abstract

This study aimed to compare the effectiveness of adsorption and photocatalysis techniques at removing the herbicide clomazone (CLO) and the antidepressant known as amitriptyline (AMI) from water. This study employed kinetic models to analyze the removal processes and assess the potential toxicity of the treated water. The structure and morphology of the prepared multi-walled carbon nanotubes were characterized as adsorbents by transmission electron microscopy, X-ray diffraction, Fourier transform infrared techniques, and Raman spectroscopy. The adsorption kinetics of CLO and AMI were studied on the pristine and functionalized multi-walled carbon nanotubes. Kinetic studies were performed by modeling the obtained experimental data using three kinetic models: pseudo-first-order, pseudo-second-order, and Elovich kinetic models. On the other hand, the efficiency of CLO and AMI photodegradation was examined as a function of the type of irradiation (UV and simulated solar irradiation) and type of TiO_2_ photocatalyst (Aeroxide and Kronos). Under the experimental conditions employed, the reaction followed pseudo-first-order kinetics. Additionally, in order to assess the toxicity of water containing CLO, AMI, and their intermediates, toxicity assessments were conducted using human fetal lung fibroblast cells. The results obtained indicate the effectiveness of both methods and provide valuable insights into their removal mechanisms, contributing to the advancement of sustainable water treatment strategies.

## 1. Introduction

The complex relationship between industrial growth and an expanding population is pervasively linked to the emission of numerous hazardous pollutants. These pollutants pose significant threats to both the ecosystem and human health. Notably, the contamination of freshwater resources has emerged as a substantial and widespread issue. The presence of dissolved pollutants, including polycyclic aromatic hydrocarbons, organic dyes, antibiotics, pesticides, heavy metals, and pharmaceutically active compounds, presents formidable challenges in terms of effectively purifying water resources [1,2,3,4,5,6,7,8].

The widespread use of pesticides, particularly herbicides, has been instrumental in inducing a significant increase in crop yields over the last few decades. This has had a direct and positive impact on food security by ensuring a consistent and abundant supply of food to meet the growing global demand [9]. However, continued reliance on these chemicals leads to ongoing damage, affecting soil quality, biodiversity, and overall ecosystem balance [10,11]. Namely, pesticides migrate through soil in different ways, and sediment and enter aquatic ecosystems through surface runoff and leaching processes, contaminating surface and groundwater [12].

Clomazone (CLO), a broad-spectrum herbicide employed to control annual grasses and broadleaf weeds in the cultivation of various crops, such as soybean, rice, tobacco, cotton, pea, maize, oilseed rape, sugar cane, cassava, and pumpkin, adds to these environmental concerns. Its physicochemical characteristics, including relatively high solubility in water (1.1 g/L), moderate mobility (*K*_OC_ = 150–562 L/kg), and persistence in soil (DT_50_ = 30–135 days), coupled with its potential adverse effects on cultivated plants during crop rotation, underscore clomazone’s potential as an agricultural environmental contaminant. Specifically, the intensive use of CLO positions this herbicide as a pollutant that may potentially affect deeper soil layers, as well as surface and groundwater, and have detrimental effects on microorganisms, plants, animals, and human beings [13,14].

As the production and use of pharmaceuticals continue to grow, their release into the environment is also on the rise. This has led to a significant increase in pharmaceutical contamination in water resources, presenting a major global environmental challenge. The aim is therefore to remove pharmaceutical contaminants from the environment, where they are even harmful at trace concentrations [15,16,17,18].

The pharmaceutical contaminant amitriptyline (AMI) is a widely utilized tricyclic antidepressant that has been on the market for an extended period of time. This pharmaceutically active compound is most commonly used in the treatment of depression, migraines, chronic pain, fibromyalgia, neurological pain, etc. Despite its higher toxicity, particularly at lower doses, than selective serotonin reuptake inhibitors, AMI continues to be widely prescribed, largely owing to its cost-effectiveness [19]. The widespread utilization of AMI has led to its frequent identification in wastewater, surface runoff, and effluent from sewage treatment plants. Consequently, there exists the potential for these contaminated waters to reach agricultural land through the utilization of municipal biosolids or reclaimed water [20].

It is becoming increasingly difficult to remove harmful pollutants from water without causing damage to the environment. This could have important implications for water treatment and environmental protection efforts [21,22]. To tackle the problem effectively, it is crucial to prioritize environmentally friendly nanomaterials that offer various benefits such as high efficiency and selectivity, low-cost production, good recyclability, and achievable stability [23,24,25,26]. Hence, numerous methods have been investigated for the removal of contaminants from aqueous media, such as adsorption [23,27,28], advanced oxidation processes [29], membrane bioreactors [24] and distillation membranes [25].

Adsorption finds diverse applications in the removal of toxic substances from water sources. This technique stands out as a cost-effective, universally applicable, and user-friendly technology for use in biological systems and wastewater treatment. Its versatility extends to the removal of both soluble and insoluble organic pollutants from diverse sources [27]. Notably, the reversible nature of the adsorption process enables the regeneration of the adsorbent, contributing to favorable operational economics [26]. Various materials, including activated carbon, chitosan, cellulose, silicates, and phyllosilicates, among others, serve as effective adsorbents [30].

Carbonaceous nanomaterials, especially activated carbon, multi-walled carbon nanotubes, single-walled carbon nanotubes, and carbon quantum dots, have been extensively researched and developed for use in various applications, particularly in wastewater treatment [22]. Since their discovery, carbon nanotubes (CNTs) have attracted considerable attention due to their distinctive properties. These properties have paved the way for potential applications across diverse domains, such as conductive and high-strength composites, nanometer-sized semiconductor devices, hydrogen storage media, and energy conversion devices [31,32]. Leveraging their large specific surface areas, hollow and layered structures, and high adsorption capacities, CNTs emerge as promising adsorbents in the realm of water treatment [33]. Thus, CNTs can be used as a promising material in environmental cleaning.

Advanced oxidation processes present innovative possibilities for transforming pollutants into non-toxic substances by generating highly oxidizing reactive oxygen species [34]. Among the various available treatment methods, photocatalysis has emerged as a pragmatic and effective solution, showcasing proven potential in the environmental cleanup of a diverse array of pollutants [7,35]. Nanostructured semiconductors, activated under solar or UV light for photocatalytic oxidation, hold great potential for use in environmental remediation [36,37].

Titanium dioxide (TiO_2_), a widely studied nanomaterial employed in environmental and energy photocatalysis, is particularly notable for its cost-effectiveness and exceptional photocatalytic activity. Different types of TiO_2_ are employed for the photodegradation of organic compounds, exhibiting distinctions in crystal composition, specific surface area, and particle size. These differences play crucial roles in determining the efficiency of TiO_2_ in the photocatalytic process [38,39,40,41,42].

Despite the potentially harmful effects of CLO and AMI on the environment, there is a lack of comprehensive scientific data regarding their efficient removal from aquatic environments. This study aimed to assess and compare the effectiveness of removing CLO and AMI from aqueous media using user-friendly techniques: adsorption in the presence of carbon nanotubes and photocatalysis performed by utilizing the commercial photocatalyst TiO_2_. The multi-walled carbon nanotubes (MWCNTs) were synthesized and characterized via transmission electron microscopy (TEM), X-ray diffraction (XRD), the Fourier Transform Infrared (FTIR) method, and Raman spectroscopy. This paper represents the first attempt at examining the adsorption kinetics of CLO and AMI utilizing the pristine (pMWCNTs) and functionalized (fMWCNTs) carbon nanotubes. The experimental data for the adsorption process were described using the Elovich, pseudo-first-order, and pseudo-second-order kinetic models. On the other hand, the photocatalytic degradation of CLO and AMI under UV light and simulated solar irradiation were investigated using two different variants of commercial TiO_2_: TiO_2_ Aeroxide (89% anatase and 11% rutile forms) and TiO_2_ Kronos (100% anatase form). In addition, the transformation products released during the photodegradation of these pollutants may be more persistent and have higher ecological or health risks than the parent substances. Thus, in order to evaluate the environmental impact of CLO, AMI and their intermediates generated during photocatalytic degradation, an in vitro toxicity assessment was performed using a human fetal lung (MRC-5) mammalian cell line. Lastly, the results achieved regarding the removal efficiency of CLO and AMI through adsorption and photodegradation were also compared.

## 2. Materials and Methods

### 2.1. Synthesis and Functionalization of MWCNTs

The synthesis of MWCNTs was conducted through the catalytic chemical vapor deposition method. This process involved a flow of ethylene/nitrogen mixture (1:1) for 1 h at 700 °C, utilizing an in situ-reduced 5% Fe-Co/Al_2_O_3_ catalyst [43] within a hand-made reactor setup, as previously outlined [44]. The resulting carbon yield was notably high, standing at 285%, and the selectivity was confirmed by the absence of amorphous carbon and other C-containing species. In order to remove the catalyst support (Al_2_O_3_), the raw material was treated in 6 mol/L NaOH under reflux at the boiling point. After washing and drying, the sample was boiled under reflux in cc. HCl for 8 h (sample marked as pMWCNTs—pristine). A part of the obtained sample was then subjected to additional acid treatment in cc. HNO_3_ for 12 h under reflux at the boiling point. This was performed to attach oxygen-containing functional groups to the sample and achieve a high degree of MWCNTs surface modification (sample marked as fMWCNTs—functionalized).

### 2.2. Characterization Methods for MWCNTs

The structure and morphology of the prepared MWCNT samples were characterized via TEM, XRD, Raman, and FTIR spectroscopy [45]. Textural characteristics were determined by employing the low-temperature N_2_ adsorption/desorption method. The specific surface area was calculated using the BET equation, while the mean pore diameter and pore volume were determined from the adsorption part of the N_2_ isotherm and calculated via the Barrett–Joyner–Halenda (BJH) method [46]. Additionally, pores were classified according to the Brunauer–Deming–Deming–Teller method [47]. The detailed procedures can be found in the Supplementary Materials.

### 2.3. Adsorption Experiments

All adsorption experiments were performed at room temperature. The kinetic experiments were carried out using 25 mL of emerging pollutant solution (CLO and AMI), with an initial concentration of 0.3 mmol/L. MWCNTs were added to solutions in the following masses: 10, 20, 30, 40, and 50 mg. During adsorption, the suspension was stirred at a constant rate. Samples were taken at defined time intervals over a period of 60 min. The obtained suspensions were filtered through Millipore (Millex-GV, 0.22 μm) PVDF membrane filters. The adsorbates were analyzed via ultrafast liquid chromatography with a diode array detector (UFLC−DAD). All the experiments were performed in duplicate.

### 2.4. Measurements of Photocatalytic Activity

The textural properties of TiO_2_ Aeroxide and TiO_2_ Kronos are presented in Table S1 (Supplementary Materials), while the major properties of investigated pollutants, which is to say herbicide CLO (CAS No 81777-89-1, 98.8%, Sigma-Aldrich, St. Louis, MO, USA) and antidepressant AMI (CAS No. 549-18-8, ≥98%, Sigma-Aldrich), are summarized in Table S2 (Supplementary Materials)). Photocatalytic experiments were performed as previously described by our group [48]. A detailed procedure can be found in the Supplementary Materials.

### 2.5. Analytical Methods

Experimental conditions for UFLC−DAD, UV energy fluxes, and pH measurements can be found in the Supplementary Materials.

### 2.6. Cytotoxic Activity

Detailed information about the cell line used in this study and growth inhibition can be found in the Supplementary Materials.

## 3. Results and Discussion

### 3.1. Characterization of Multi-Walled Carbon Nanotubes

**Figure 1:**
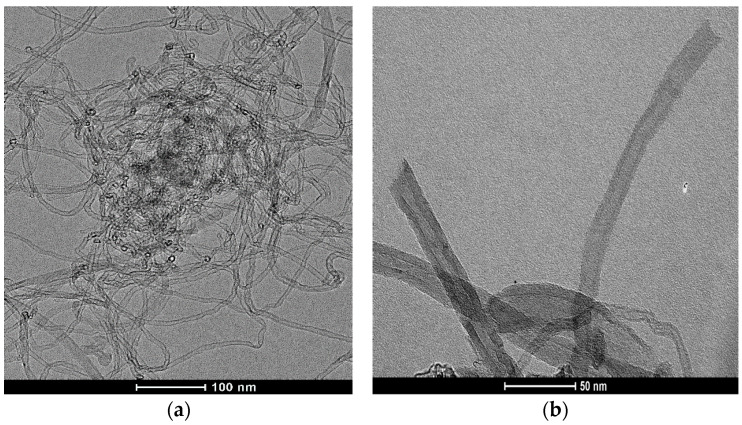
TEM images of (**a**) pMWCNTs and (**b**) fMWCNTs.

The TEM images of pristine MWCNTs (Figure 1a) testified to a very high aspect ratio and the highly warped and interwoven tube structures that formed a dense network, which is typical for such a material [49]. Many of the nanotubes (90%) had external diameters, which mostly ranged within 5–30 nm. We detected that no traces of catalyst remained in the formed tubes. Also, the presence of amorphous carbon and other non-selective carbon species instead of MWCNTs was not identified via TEM analysis. After the applied oxidation treatment in cc. HNO_3_, most of the nanotubes were opened at their ends (Figure 1b). Structural alterations and the extent of functionalization have frequently been observed to correlate with the concentration of the acid utilized during liquid oxidation and with the duration of the treatment [50].

**Figure 2:**
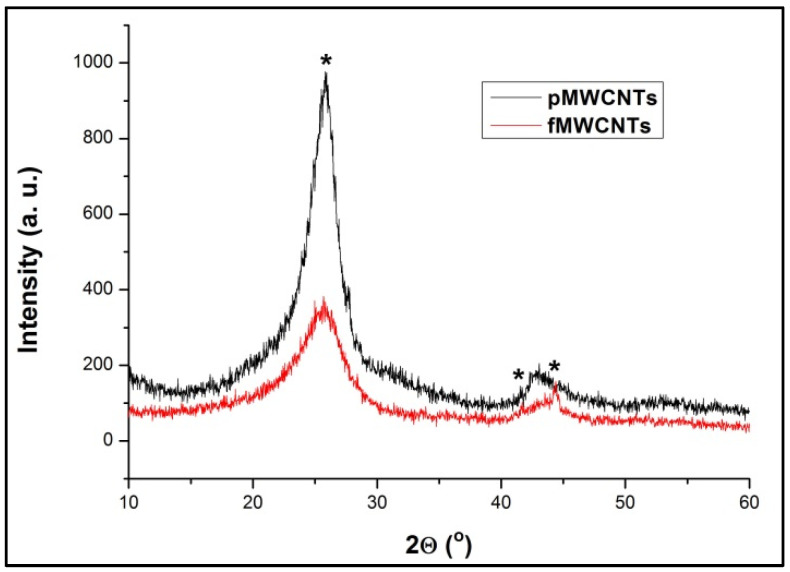
XRD patterns of pMWCNTs and fMWCNTs (the characteristic peaks are marked with *).

As can be seen from Figure 2, both patterns exhibit characteristic Bragg’s reflections, indicative of crystalline graphite. The most prominent peak appears sharply at 2*θ* = 26° (*002*), and a broader peak around 2*θ* = 43° is formed by the overlapping signals of (*010*) and (*011*) (COD database code: 1011060, reference code 96-101-1061). Prior to performing XRD parameter analysis, the profiles of the (*002*) peak were fitted using the pseudo-Voigt function. The resulting values for inter-layer distance (d_002_) (Bragg’s law) were remarkably similar for both samples, hovering around 0.34 nm, while the mean diameters of crystallites along the nanotube diameter (Debye-Scherrer’s equation) were 2.9 nm and 1.8 nm for pMWCNTs and fMWCNTs, respectively, revealing their reduction to some extent following oxidation treatment. Additionally, the decrease in the (*002*) peak intensity for the fMWCNTs sample might be an indication of the deterioration of the tube graphitization degree (order of crystallinity) due to the introduction of functional groups.

**Figure 3:**
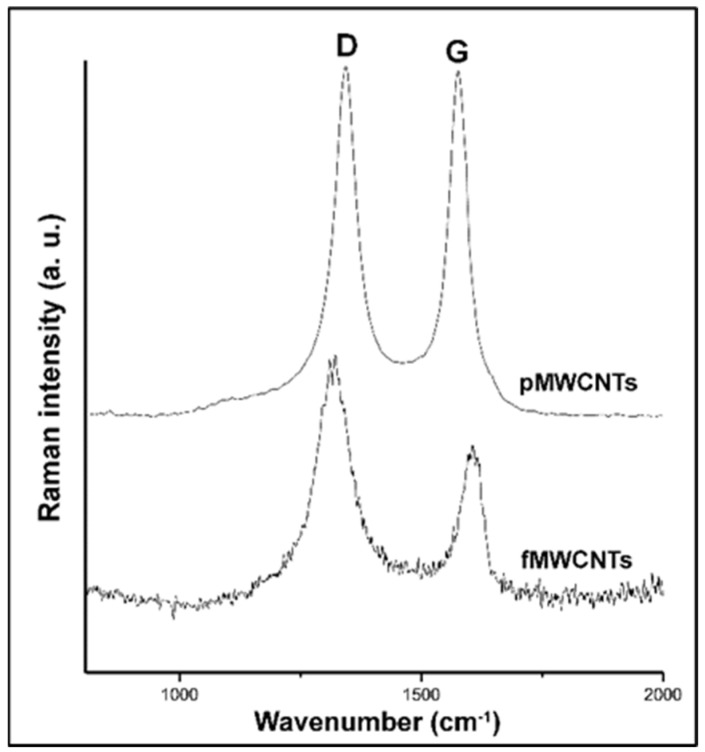
Raman spectra of pMWCNTs and fMWCNTs.

As a well-known tool used to characterize sp^2^ and sp^3^ hybridized carbon atoms, Raman spectroscopy was used to differentiate the structure of the examined MWCNT samples. Figure 3 shows two prominent Raman features observed in the spectra of both samples. Their peaks are situated around 1340 cm^−1^ and 1580 cm^−1^, corresponding to the D and G bands, respectively [51]. The conventional interpretations of these bands are linked to the structural defects present in the graphitic tube walls (D band) and the high symmetry of ordered multi-walled carbon nanotubes (G band).

In order to compare the structural quality of the MWCNTs in terms of the density of the present defects, the Raman quality indicator, the I_D_/I_G_ ratio, was calculated as the ratio of the integrated areas of these bands [52]. The significantly higher I_D_/I_G_ ratio (1.76) obtained for the fMWCNTs compared to the pMWCNTs (1.03) indicates increased defect density after oxidation treatment. Additionally, the crystalline quality deterioration of the fMWCNTs, designated by the broadening of the D and G peaks, fits into the same picture produced by XRD analysis.

**Figure 4:**
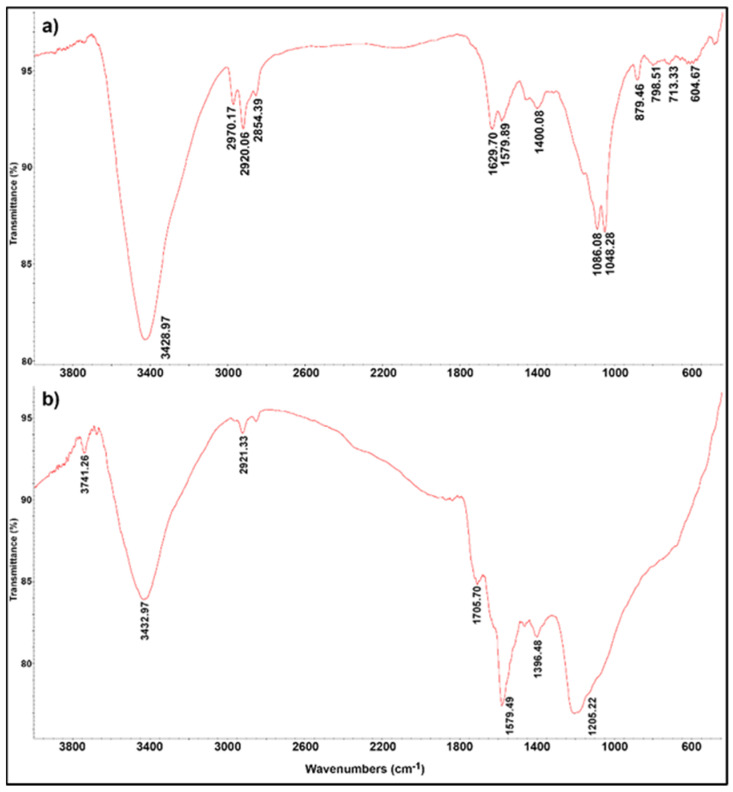
FTIR spectra of: (**a**) pMWCNTs; (**b**) fMWCNTs.

The extent of the surface modification of the synthesized carbon nanotubes upon liquid oxidation treatment was examined using FTIR spectroscopy (Figure 4). Both samples could be characterized by broad and intensive vibrational bands at ~3400 cm^−1^, which could be attributed to the stretching vibrations of –OH groups. These groups could be directly attached to the surface of the tubes and/or originated from carboxylic groups and adsorbed water molecules (moisture) [53]. The stretching vibrations of –CH_2_ groups at ~2920 cm^−1^ (asymmetric –C–H stretching vibrations—aliphatic), as well as the stretching vibrations of –CH_3_ groups at ~2850 cm^−1^ (symmetric –C–H stretching vibrations—aliphatic), were also present in both samples, while the band at ~1630 cm^−1^, observable in pMWCNTs, originated mainly from the water adsorbed onto the KBr and MWCNTs [54]. The band at ~1580 cm^−1^, displaying emphasized intensity in the case of fMWCNTs, was associated with the vibrations of –C=O groups [55]. Additionally, the presence of carboxylic and/or carbonyl groups in the structure of fMWCNTs was indicated by the peak at ~1705 cm^−1^, corresponding to the stretching vibrations of –C=O groups [56]. The absorption band at ~1400 cm^−1^, characteristic of MWCNT samples, signified the deformation of the –C–H bond and acted as proof of the presence of –CH_3_ groups [57]. The functionalized sample was also characterized by a broad band at ~1200 cm^−1^, which could be assigned to –C–O– stretching and –OH bending from carboxylic groups, while the two bands at 1086 cm^−1^ and 1048 cm^−1^ in the spectra of pMWCNTs were the consequence of –C–O– stretching in alcoholic compounds [58]. The presented results of FTIR analysis indicated that a very small amount of oxygenated functional groups was present in the structure of pMWCNTs, while the surface of fMWCNTs was enriched with –OH, –C=O, –COOH groups. Boehm titration was used to quantify acidic and basic groups on the surfaces of both samples. According to the results of Boehm titration (Table 1), the fMWCNTs contained a much higher amount of oxygenated acidic functional groups compared to their pristine counterpart, with dominating phenolic groups contributing to the total acidity of the sample, at 79.7%. The portion of carboxylic groups was much lower (19.4%), while lactone groups only had a 0.9% presence. The presence of basic functional groups was not detected in any of the examined MWCNTs.

**Figure 5:**
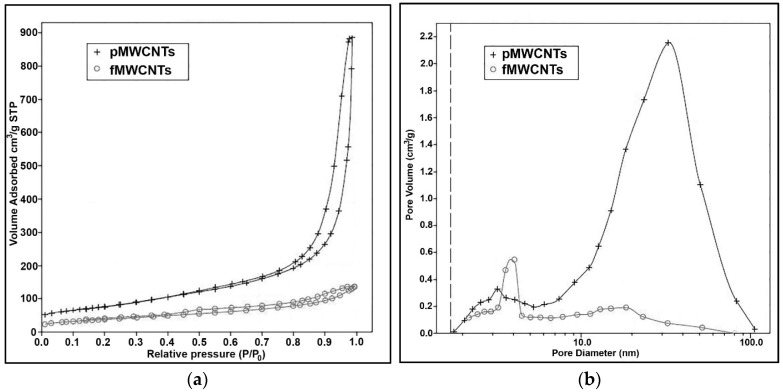
(**a**) N_2_ adsorption–desorption isotherms; (**b**) BJH pore–size distributions from the desorption parts of the isotherms for MWCNTs.

The N_2_ adsorption–desorption isotherms and BJH pore–size distributions for the MWCNT samples are illustrated in Figure 5 and detailed in Table 2. The isotherms (Figure 5a) for both samples exhibit H_3-_type hysteresis loops, indicative of the presence of slit-shaped mesopores [47]. The pMWCNTs possess a high specific surface area (272.5 m^2^/g) and total pore volume (1.4 mL/g), while the obtained parameters for the fMWCNTs indicate the changes in textural properties caused by surface modification. Namely, all the parameters show decreases after oxidation treatment. pMWCNTs are characterized by a highly developed mesoporosity (bimodal pore–size distribution profile), with most pores measuring approximately 30 nm in diameter (Figure 5b). They are predominately formed within the confined spaces among isolated nanotubes. After functionalization, the proportion of these pores decreases significantly, which might be the consequence of the emphasized attractive interactions between oxygen-containing functional groups attached to the surface of the tubes, causing their structural reorganization. The mesopores in a lower diameter range (2–5 nm) can also be observed in both samples; however, the increase in their fraction in fMWCNTs is in accordance with the results of TEM analysis, revealing the opening of some nanotubes after the introduction of oxygen-containing functional groups.

### 3.2. Adsorption and Kinetic Models and of CLO and AMI on MWCNTs

In order to investigate the rate of the adsorption uptake of CLO and AMI to the pMWCNTs and fMWCNTs, kinetic experiments were conducted and the obtained data were fitted using kinetic models: pseudo-first-order and pseudo-second-order kinetic models and the Elovich equation. The nonlinear forms of kinetic equations were used [59,60]. The Elovich model has been used to fit kinetic data for the adsorption of CLO [61], while the use of pseudo-first- and pseudo-second-order models has been reported for fitting the kinetic data for the adsorption of AMI to carbonaceous material [62]. The obtained kinetic parameters are presented in Table 3.

In the case of CLO, the average values of the correlation coefficient for the adsorption onto both pMWCNTs and fMWCNTs had the highest value for the pseudo-second-order model (0.996 and 0.996), followed by the pseudo-first-order model (0.994 and 0.996). Both models were good at representing clomazone’s adsorption onto multi-walled nanotubes. Overall, we observed lower values of adsorbed amounts at the equilibrium of the adsorption of clomazone onto the fMWCNTs than onto the pMWCNTs. This indicated that the increase in the acidity of the surface of the adsorbent and the lower specific surface negatively influenced the adsorption of clomazone. Clomazone has more polar groups than amitriptyline, indicating stronger electrostatic interactions with the surface. A combination of electrostatic interactions and the lower specific surface with smaller pores could explain the lower amounts of clomazone adsorbed onto the fMWCNTs.

Based on the correlation coefficient values, the adsorption of AMI onto pMWCNTs is best represented by the Elovich model, while the kinetics of adsorption onto the fMWCNTs is better fitted by the pseudo-second-order model. This could indicate the different mechanisms between the adsorption of AMI onto the pMWCNTs and the fMWCNTs. The adsorbed amounts of amitriptyline at the equilibrium are overall larger for adsorption onto the fMWCNTs than onto the pMWCNTs. A possible explanation for this is that, although pMWCNTs have larger specific surfaces, the larger value of the total acidity of the fMWCNTs could have an impact on the adsorption process of the AMI. This would probably be due to the presence of the amino group in the molecule.

**Figure 6:**
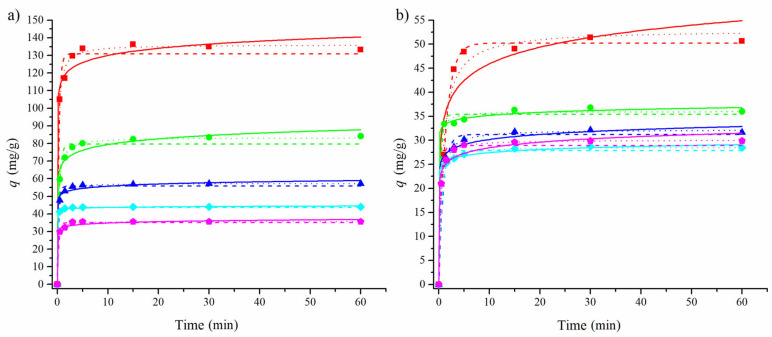
Experimental data for the adsorption of CLO (0.3 mmol/L) onto the pMWCNTs (**a**) and fMWCNTs (**b**), fitted with pseudo-first-order (---), pseudo-second-order (···), and Elovich (–) models. Different masses of adsorbents were used in the experiments: 10 (◼), 20 (●), 30 (▲), 40 (◆), and 50 (⬟) mg.

**Figure 7:**
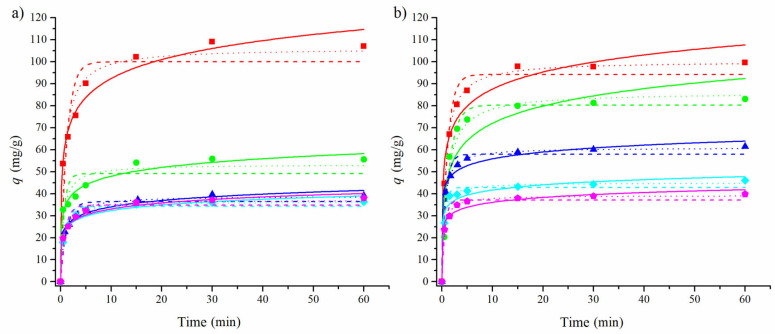
Experimental data for the adsorption of AMI (0.3 mmol/L) onto the pMWCNTs (**a**) and fMWCNTs (**b**), fitted with pseudo-first-order (---), pseudo-second-order (···), and Elovich (–) models. Different masses of adsorbents were used in the experiments: 10 (◼), 20 (●), 30 (▲), 40 (◆), and 50 (⬟) mg.

A visual inspection of the fitted data for both adsorbates (Figure 6 and Figure 7) shows that the Elovich model tends to predict larger values of the adsorbed amounts for the points close to the equilibrium than for the experimentally obtained data.

### 3.3. Photodegradation of CLO and AMI Using TiO_2_ Kronos and TiO_2_ Aeroxide

#### 3.3.1. Kinetics of Photocatalytic Degradation of CLO and AMI 

The present study investigated the photocatalytic activity of two types of TiO_2_ photocatalysts (Aeroxide and Kronos) in the removal of CLO and AMI. In order to compare the types of irradiation, the photodegradation efficiency of the mentioned herbicide and antidepressant were also tested under UV light and simulated solar irradiation. The kinetics of the photocatalytic degradation of CLO and AMI are described by the Langmuir–Hinshelwood model. Apparent rate constants, *k*_app_, and the coefficient of determination, *R*^2^, were calculated with linear fitting [63]. The obtained data revealed that practically no degradation of CLO and AMI was observed under conditions of simulated solar irradiation after 120 min of the process, rendering the determination of a rate constant unfeasible. Namely, under the same length of time, the removal activity of TiO_2_ Aeroxide and TiO_2_ Kronos showed that these nanopowders were almost inactive regarding the removal of CLO and AMI. However, the removal efficiency of CLO and AMI differed to a higher extent under UV irradiation in the presence of TiO_2_ Kronos and TiO_2_ Aeroxide, where the system with TiO_2_ Aeroxide proved to be significantly more efficient. The results obtained for *k*_app_ when using TiO_2_ Kronos and TiO_2_ Aeroxide as photocatalysts under UV conditions are given in Table 4. It was found that *k*_app_ was significantly increased when the process was catalyzed by TiO_2_ Aeroxide compared to TiO_2_ Kronos. This behavior can be explained by the differences in the morphologies of the photocatalysts (Table S1, Supplementary Materials). Namely, the type of photocatalyst has a significant influence on the efficiency of CLO and AMI photocatalytic degradation. Moreover, this study found that the photodegradation efficiency of the herbicide CLO was higher than that of AMI, as indicated by the obtained values of *k*_app_ for CLO removal (Table 4). Overall, the research emphasizes the significance of both photocatalyst type and irradiation conditions in determining the efficacy of photocatalytic degradation processes for CLO and AMI.

#### 3.3.2. In Vitro Cytotoxicity Assay

Since TiO_2_ Aeroxide proved to be the most efficient nanopowder when using UV light, this catalyst was used in the further examination of the toxicity of CLO and AMI, as well as the toxicity of their intermediates formed during the photocatalytic process. The assessment of cytotoxic activity involved the application of the modified MTT assay, with MRC-5 samples subjected to a 48 h treatment. The cytotoxicity testing results are given in Table 5.

Based on the obtained results (Table 5), it can be found that the stock solution of CLO showed a moderately low cytotoxicity (IC_50_ = 50.5 μM), while the stock solution of AMI showed a weak cytotoxicity (IC_50_ = 87.6 μM). However, the IC_50_ values of samples obtained after 45 min of UV irradiation using TiO_2_ Aeroxide (CLO/TiO_2_ Aeroxide/UV and AMI/TiO_2_ Aeroxide/UV) were >100 μM. Namely, the UV light and photocatalyst significantly reduced the toxicity of CLO and AMI. Also, these findings indicated that the obtained intermediates were not toxic to MRC-5. In our previous study [48], similar results were obtained regarding the photodegradation of CLO using TiO_2_ Degussa P25 (75% anatase and 25% rutile forms) under UV irradiation. The analysis of intermediate products generated during the process revealed the formation of various organic intermediates and ionic byproducts during irradiation. Comparing the obtained results of cytotoxicity towards both the H-4-II-E and MRC-5 cell lines, as well as the degradation kinetics, it was found that irradiation contributed to a decrease in the toxicity of the mixture, which was no longer dominated by the CLO pollutant.

**Figure 8:**
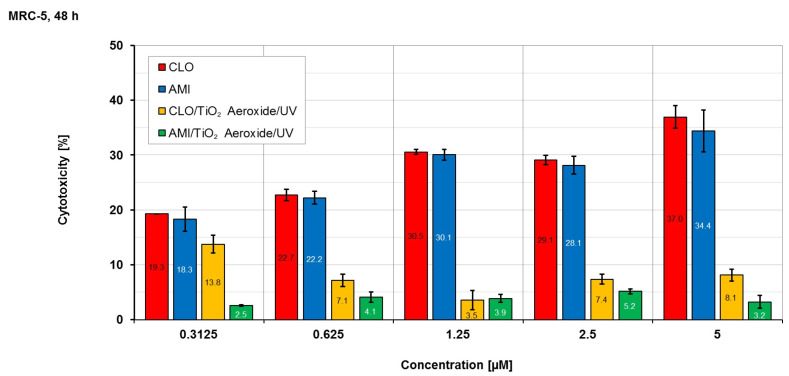
The cytotoxicity results after 48 h of treatment of MRC-5 cultures with different concentrations.

The cytotoxicity results obtained for samples of MRC-5 cells treated using the array of applied concentrations are shown in Figure 8.

Analyzing the results (Figure 8), it can be noted that the measured cytotoxicity of pollutants CLO and AMI appears to be linear and dose-dependent. On the other hand, the response of intermediates obtained in the CLO/TiO_2_ Aeroxide/UV and AMI/TiO_2_ Aeroxide/UV systems was low, non-linear, and not related to the concentrations used. Such a response could be described as displaying low activity combined with a hormetic effect.

### 3.4. Assessing the Removal Efficiency of CLO and AMI through Adsorption and Photodegradation

Important elements in the application of water treatment methods include information about the achieved degree of pollutant removal during the process. In the present study, the efficiency of CLO and AMI removal from water was evaluated and compared using adsorption and photocatalysis.

**Figure 9:**
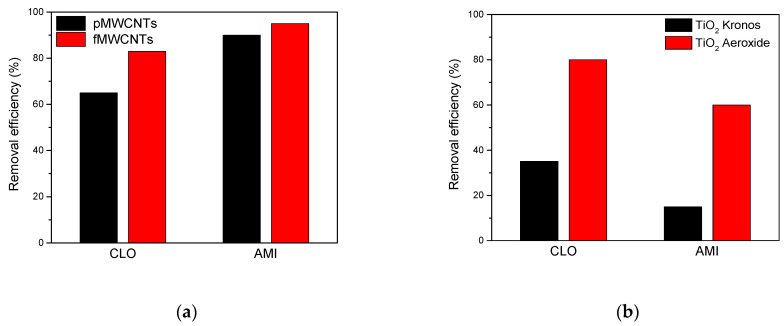
The removal efficiency of CLO and AMI (0.3 mmol/L) from water: (**a**) after 45 min adsorption using 2.0 mg/mL adsorbent; (**b**) after 45 min irradiation under UV in the presence of 2.0 mg/mL photocatalyst.

The results presented in Figure 9a show the significant adsorption of CLO and AMI onto the MWCNTs surface. In the case of pMWCNTs, the rate of adsorption for herbicide was around 65%, whereas it was 83% for fMWCNTs. Interestingly, AMI showed significantly higher adsorption when using MWCNTs as an adsorbent. Under the same conditions (during 45 min), the pMWCNTs removed 90% while the FMWCNTs removed 95% of AMI (Figure 9a).

In the context of TiO_2_ Kronos/UV and TiO_2_ Aeroxide/UV, approximately 35% and 80% of CLO was removed, respectively, after a 45 min process (Figure 9). This study also examined the photocatalytic degradation efficiency of AMI under UV irradiation, utilizing TiO_2_ Kronos and TiO_2_ Aeroxide. The results presented in Figure 9b demonstrate the significantly higher efficiency of AMI degradation in the presence of TiO_2_ Aeroxide compared to TiO_2_ Kronos. After 45 min of irradiation, 15% and 60% of AMI was removed in the presence of TiO_2_ Kronos and TiO_2_ Aeroxide, respectively. This observed behavior can be attributed to disparities in the morphologies of the photocatalysts (Table S1, Supplementary Materials).

In summary, adsorption emerged as an effective technique for removing the specified pollutants from water, particularly in the case of AMI. Nevertheless, it is crucial to recognize that water purification through physical methods like adsorption onto activated carbon involves the transfer of pollutants from one phase to another, incurring additional recycling costs. Despite the effectiveness of photocatalytic decomposition of CLO and AMI being dependent on the radiation source, the utilization of photocatalytic degradation in treating polluted water offers advantages by transforming the herbicide and antidepressant into non-toxic compounds. This indicates the need for further research in order to investigate the practicality and efficacy of adsorption and photocatalysis in eliminating CLO and AMI contaminants from water bodies, thus contributing to safeguarding aquatic ecosystems and their biodiversity. Additional considerations may include economic cost-effectiveness, environmental impacts, and practical implementation in real conditions, etc.

## 4. Conclusions

Pristine and functionalized multi-walled carbon nanotubes were synthesized and characterized using TEM, XRD, FTIR, and Raman spectroscopy. Kinetic studies were conducted for the adsorption of CLO and AMI from aqueous solutions. The results indicated that the adsorption kinetics of CLO onto adsorbents (pMWCNTs and fMWCNTs) followed the pseudo-second-order and pseudo-first-order models. The adsorption kinetics of AMI onto fMWCNTs exhibited a better fit with the pseudo-second order model, while the adsorption of AMI on pMWCNTs was best represented by the Elovich model. In the case of the kinetics of photocatalytic degradation CLO and AMI, the TiO_2_ system known as Aeroxide/UV was identified as the most efficient and was chosen to undergo further investigations. Namely, the photocatalytic degradation of CLO and AMI using TiO_2_ Aeroxide/UV followed pseudo-first-order kinetics. Furthermore, the toxicity assessment of mixtures of AMI and CLO, as well as respective photocatalytic degradation intermediates produced using TiO_2_ Aeroxide under UV irradiation conditions did not show any significant impact on the MRC-5 cell line.

In the context of pMWCNTs, the adsorption of herbicide was around 65%, while it was 83% for fMWCNTs. Moreover, under the same conditions, pMWCNTs removed 90%, while FMWCNTs removed 95%, of AMI. In the instance of TiO_2_ Kronos/UV and TiO_2_ Aeroxide/UV, approximately 35% and 80% of CLO was removed, respectively. After 45 min of irradiation, 15% and 60% of AMI was removed in the presence of TiO_2_ Kronos and TiO_2_ Aeroxide, respectively.

## Figures and Tables

**Table 1:** The results of Boehm titration.

Sample	Functional Groups				
	Acidic Sites (mmol/g)				Basic Sites (mmol/g)
	Carboxylic	Lactonic	Phenolic	Total acidity	
pMWCNTs	0.03	0.01	0.81	0.85	-
fMWCNTs	0.45	0.02	1.85	2.32	-

**Table 2:** Textural properties of MWCNTs.

Sample	Textual Property		
	BET (m^2^/g)	Average Pore Diameter (nm)	Total Pore Volume (mL/g)
pMWCNTs	272.5	15.7	1.4
fMWCNTs	136.9	5.8	0.2

**Table 3:** Kinetic parameters obtained by fitting experimental data for the adsorption of CLO and AMI on pMWCNTs and fMWCNTs using pseudo-first-order, pseudo-second-order, and Elovich models.

			Pseudo-First-Order Model			Pseudo-Second-Order Model			Elovich Model		
Adsorbate	Adsorbent	*m_ads_* (mg)	*q_e_*_I_ (mg/g)	*k*_I_ (min^−1^)	*R* ^2^	*q_e_*_II_ (mg/g)	*k*_II_ (g/(mg min))	*R* ^2^	*α* (mg/(g min))	*β* (mg/g)	*R* ^2^
**CLO**	**pMWCNTs**	10	130.86	3.122	0.985	136.09	0.045	0.996	3.029 × 10^9^	0.172	0.984
		20	79.73	2.623	0.996	83.43	0.058	0.999	1.208 × 10^7^	0.215	0.985
		30	55.88	3.801	0.996	57.31	0.169	0.999	8.397 × 10^12^	0.565	0.993
		40	43.71	5.833	0.999	44.08	0.687	0.999	2.062 × 10^39^	2.145	0.999
		50	35.04	3.750	0.992	35.83	0.264	0.983	4.903 × 10^12^	0.903	0.990
	**fMWCNTs**	10	50.23	0.732	0.999	52.98	0.023	0.986	4838.37	0.200	0.937
		20	35.41	2.710	0.995	36.01	0.265	0.996	1.009 × 10^16^	1.116	0.998
		30	31.20	1.126	0.994	32.25	0.086	0.999	8.693 × 10^7^	0.669	0.994
		40	27.88	1.514	0.998	28.60	0.161	0.999	6.375 × 10^11^	1.078	0.999
		50	28.93	2.427	0.988	30.07	0.150	0.998	3.806 × 10^6^	0.595	0.980
**AMI**	**pMWCNTs**	10	99.93	0.807	0.906	106.20	0.013	0.970	2091.86	0.080	0.984
		20	49.07	1.441	0.972	53.30	0.034	0.936	2571.26	0.175	0.985
		30	36.42	0.851	0.941	38.98	0.034	0.987	1542.52	0.242	0.990
		40	34.21	1.117	0.998	36.31	0.049	0.999	2401.26	0.272	0.968
		50	34.80	1.117	0.936	37.11	0.047	0.899	1841.14	0.256	0.987
	**fMWCNTs**	10	94.16	0.919	0.968	100.19	0.014	0.998	2789.45	0.089	0.966
		20	80.28	0.706	0.993	86.03	0.012	0.980	641.93	0.088	0.899
		30	57.88	1.302	0.980	60.90	0.039	0.999	1.153 × 10^6^	0.223	0.986
		40	42.80	1.857	0.998	44.93	0.069	0.995	82060.74	0.298	0.969
		50	37.15	1.662	0.967	39.18	0.069	0.969	25894.01	0.313	0.978

**Table 4:** Apparent rate constants (*k*_app_) determined for 45 min irradiation using UV. Results are expressed as mean ± SD of three independent experiments.

	*k*_app_ (min^−1^)	*R* ^2^	*k*_app_ (min^−1^)	*R* ^2^
Pollutant	TiO_2_ Kronos		TiO_2_ Aeroxide	
CLO	0.0038	0.963	0.027	0.995
AMI	0.0016	0.626	0.018	0.957

**Table 5:** IC_50_ * (μM) values after 48 h of treatment of MRC-5 cells, obtained with the MTT test.

Sample	MRC-5
CLO	50.5
AMI	87.6
CLO/TiO_2_ Aeroxide/UV	>100
AMI/TiO_2_ Aeroxide/UV	>100
Ultrapure water	N/A

* IC_50_ is the concentration of compound required to inhibit cell growth by 50% compared to an untreated control. The coefficients of variation were less than 10%.

## Data Availability

Data are contained within the article.

## Supplementary Materials

The following supporting information can be downloaded at: https://www.mdpi.com/article/10.3390/ma17061369/s1, Table S1: Structural parameters of the investigated photocatalysts; Table S2: Physicochemical properties of investigated pollutants.

## References

[1] Zhang Z., Jiatieli J., Liu D., Yu F., Xue S., Gao W., Li Y., Dionysiou D.D. (2013). Microwave induced degradation of parathion in the presence of supported anatase- and rutile-TiO_2_/AC and comparison of their catalytic activity. Chem. Eng. J..

[2] Zeng G., Chen M., Zeng Z. (2013). Risks of neonicotinoid pesticides. Science.

[3] Gómez-Alvarez M., Poznyak T., Ríos-Leal E., Silva-Sánchez C. (2012). Anthracene decomposition in soils by conventional ozonation. J. Environ. Manag..

[4] Jamieson A.J., Malkocs T., Piertney S.B., Fujii T., Zhang Z. (2017). Bioaccumulation of persistent organic pollutants in the deepest ocean fauna. Nat. Ecol. Evol..

[5] Rice J., Westerhoff P. (2017). High levels of endocrine pollutants in US streams during low flow due to insufficient wastewater dilution. Nat. Geosci..

[6] Fujita M., Ide Y., Sato D., Kench P.S., Kuwahara Y., Yokoki H., Kayanne H. (2014). Heavy metal contamination of coastal lagoon sediments: Fongafale Islet, Funafuti Atoll, Tuvalu. Chemosphere.

[7] Zangeneh H., Zinatizadeh A.A.L., Habibi M., Akia M., Hasnain Isa M. (2015). Photocatalytic oxidation of organic dyes and pollutants in wastewater using different modified titanium dioxides: A comparative review. J. Ind. Eng. Chem..

[8] Shajahan S., Mohammad A.H. (2023). Development of Co_3_O_4_/TiO_2_/rGO photocatalyst for efficient degradation of pharmaceutical pollutants with effective charge carrier recombination suppression. Environ. Res..

[9] Tilman D., Cassman K.G., Matson P.A., Naylor R., Polasky S. (2002). Agricultural sustainability and intensive production practices. Nature.

[10] Manz M., Wenzel K.D., Dietze U., Schüürmann G. (2001). Persistent organic pollutants in agricultural soils of central Germany. Sci. Total Environ..

[11] Cheng M., Zeng G., Huang D., Lai C., Xu P., Zhang C., Liu Y. (2016). Hydroxyl radicals based advanced oxidation processes (AOPs) for remediation of soils contaminated with organic compounds: A review. Chem. Eng. J..

[12] Li R., Hu W., Liu H., Huang B., Jia Z., Liu F., Zhao Y., Khan K.S. (2024). Occurrence, distribution, and ecological risk assessment of herbicide residues in cropland soils from the Mollisols region of Northeast China. J. Hazard. Mater..

[13] MacBean C. (2012). The Pesticide Manual, A World Compendium.

[14] Andres A., Concenço G., Theisen G., Vidotto F., Ferrero A. (2013). Selectivity and weed control efficacy of pre- and post-emergence applications of clomazone in Southern Brazil. Crop Prot..

[15] Patel M., Kumar R., Kishor K., Mlsna T., Pittman C.U., Mohan D. (2019). Pharmaceuticals of emerging concern in aquatic systems: Chemistry, occurrence, effects, and removal methods. Chem. Rev..

[16] O’Flynn D., Lawler J., Yusuf A., Parle-Mcdermott A., Harold D., Mc Cloughlin T., Holland L., Regan F., White B. (2021). A review of pharmaceutical occurrence and pathways in the aquatic environment in the context of a changing climate and the COVID-19 pandemic. Anal. Methods.

[17] Maculewicz J., Kowalska D., Świacka K., Tónski M., Stepnowski P., Białk-Bielinska A., Dołzonek J. (2022). Transformation products of pharmaceuticals in the environment: Their fate, (eco)toxicity and bioaccumulation potential. Sci. Total Environ..

[18] Vaudin P., Augé C., Just N., Mhaouty-Kodja S., Mortaud S., Pillon D. (2022). When pharmaceutical drugs become environmental pollutants: Potential neural effects and underlying mechanisms. Environ. Res..

[19] Nabais J.M.V., Ledesma B., Laginhas C. (2012). Removal of amitriptyline from aqueous media using activated carbons. Adsorpt. Sci. Technol..

[20] Lv G., Stockwell C., Niles J., Minegar S., Li Z., Jiang W. (2013). Uptake and retention of amitriptyline by Kaolinite. J. Colloid Interface Sci..

[21] Al-Ghouti M.A., Maryam A.A., Mohammad Y.A., Dana A.D. (2019). Produced water characteristics, treatment and reuse: A review. J. Water Proc. Eng..

[22] Raza A., Altaf S., Ali S., Ikram M., Li G. (2022). Recent advances in carbonaceous sustainable nanomaterials for wastewater treatments. Sustain. Mater. Technol..

[23] Kolahalam L.A., Kasi Viswanath I.V., Diwakar B.S., Govindh B., Reddy V., Murthy Y.L.N. (2019). Review on nanomaterials: Synthesis and applications. Mater. Today Proc..

[24] Govan J. (2020). Recent Advances in Magnetic Nanoparticles and Nanocomposites for the Remediation of Water Resources. Magnetochemistry.

[25] Tahir M.B., Sohaib M., Sagir M., Rafique M. (2020). Role of Nanotechnology in Photocatalysis. Encyclopedia of Smart Materials.

[26] Nadeem M.S., Munawar T., Mukhtar F., Rabbani A.W., Khan S.A., Koc M., Iqbal F. (2023). Synergistic photocatalytic properties of fullerene (C60) anchored V/Cu dual-doped NiO nanocomposites for water disinfection. Mater. Sci. Eng. B.

[27] Akinpelu A.A., Ali M.E., Johan M.R., Saidur R., Qurban M.A., Saleh T.A. (2019). Polycyclic aromatic hydrocarbons extraction and removal from wastewater by carbon nanotubes: A review of the current technologies, challenges and prospects. Process Saf. Environ. Prot..

[28] Hassanpour M., Safardoust-Hojaghan H., Salavati-Niasari M. (2017). Degradation of methylene blue and Rhodamine B as water pollutants via green synthesized Co_3_O_4_/ZnO nanocomposite. J. Mol. Liq..

[29] Yaseen D.A., Scholz M. (2019). Textile dye wastewater characteristics and constituents of synthetic effluents: A critical review. Int. J. Environ. Sci. Technol..

[30] Alencar J.M., Oliveira F.J.V.E., Airoldi C., da Silva Filho E.C. (2014). Organophilic nickel phyllosilicate for reactive blue dye removal. Chem. Eng. J..

[31] Baughman R.H., Zakhidov A.A., de Heer W.A. (2002). Carbon nanotubes–the route toward applications. Science.

[32] Zhang S., Shao T., Bekaroglu S.S.K., Karanfil T. (2010). Adsorption of synthetic organic chemicals by carbon nanotubes: Effects of background solution chemistry. Water Res..

[33] Upadhyayula V.K.K., Deng S.G., Mitchell M.C., Smith G.B. (2009). Application of carbon nanotube technology for removal of contaminants in drinking water: A review. Sci. Total Environ..

[34] Dong C., Yang Y., Hu X., Cho Y., Jang G., Ao Y., Wang L., Shen J., Park J.H., Zhang K. (2022). Self-cycled photoFenton-like system based on an artificial leaf with a solar-to-H_2_O_2_ conversion efficiency of 1.46%. Nat. Commun..

[35] Reddy P.A.K., Reddy P.V.L., Kwon E., Kim K.-H., Akter T., Kalagar S. (2016). Recent advances in photocatalytic treatment of pollutants in aqueous media, Review article. Environ. Inter..

[36] Feizpoor S., Habibi-Yangjeh A. (2018). Ternary TiO_2_/Fe_3_O_4_/CoWO_4_ nanocomposites: Novel magnetic visible-light-driven photocatalysts with substantially enhanced activity through pn heterojunction. J. Colloid Interface Sci..

[37] Raza A., Zhang Y., Cassinese A., Li G. (2022). Engineered 2D Metal Oxides for Photocatalysis as Environmental Remediation: A Theoretical Perspective. Catalysts.

[38] Zhao T., Zhou Q., Lv Y., Han D., Wu K., Zhao L., Shen Y., Liu S., Zhang Y. (2020). Ultrafast condensation of carbon nitride on electrodes with exceptional boosted photocurrent and electrochemiluminescence. Angew. Chem. Int Ed. Engl..

[39] Xia H., Xu X., Li D. (2022). Ligand-Decomposition assistant formation of CdS/TiO_2_ hybrid nanostructure with enhanced photocatalytic activity. J. Alloys Compd..

[40] Jiang R., Zhu W., Li K., Zhu W., Ye G., Jia G., Xu F., Wang J., Tao T., Wang Y. (2022). TiO_2_/*β*-C_3_N_4_ for sunlight-driven overall water splitting. J. Alloys Compd..

[41] Wang T., Zhu Q., Huo C., Yin Z., Shi Q., Tao J., Su F., Cao S. (2023). Constructing flower-like TiO_2_/Bi_2_O_3_ p-n heterojunction with enhanced visible-light photocatalytic performance. J. Alloys Compd..

[42] Guillard C., Puzenat E., Lachheb H., Houas A., Herrmann J.M. (2005). Why inorganic salts decrease the TiO_2_ photocatalytic efficiency. Int. J. Photoenergy.

[43] Panic S., Rakic D., Guzsvány V., Kiss E., Boskovic G., Kónya Z., Kukovecz A. (2015). Optimization of thiamethoxam adsorption parameters using multi-walled carbon nanotubes by means of fractional factorial design. Chemosphere.

[44] Ratkovic S., Kiss E., Boskovic G. (2009). Synthesis of high-purity carbon nanotubes over alumina and silica supported bimetallic catalysts. Chem. Ind. Chem. Eng. Q..

[45] Boehm H.P. (2002). Surface oxides on carbon and their analysis: A critical assessment. Carbon.

[46] Barrett E.P., Joyner L.G., Halenda P.P. (1951). The Determination of Pore Volume and Area Distributions in Porous Substances. I. Computations from Nitrogen Isotherms. J. Am. Chem. Soc..

[47] Lowell S., Shields J.E., Thomas M.A., Thommes M. (2004). Characterization of Porous Solids and Powders: Surface Area, Pore Size and Density.

[48] Abramović B.F., Despotović V.N., Sojić D.V., Orcić D.Z., Csanadi J.J., Cetojević-Simin D.D. (2013). Photocatalytic degradation of the herbicide clomazone in natural water using TiO_2_: Kinetics, mechanism, and toxicity of degradation products. Chemosphere.

[49] Panic S., Bajac B., Rakić S., Kukovecz Á., Kónya Z., Srdić V., Boskovic G. (2017). Molybdenum anchoring effect in Fe-Mo/MgO catalyst for multiwalled carbon nanotube synthesis. React. Kinet. Mech. Catal..

[50] Kanyó T., Kónya Z., Kukovecz Á., Berger F., Dékány I., Kiricsi I. (2004). Quantitative Characterization of Hydrophilic-Hydrophobic Properties of MWNTs Surfaces. Langmuir.

[51] Dresselhaus M.S., Dresselhaus G., Saito R., Jorio A. (2005). Raman spectroscopy of carbon nanotubes. Phys. Rep..

[52] Ferrari A.C., Robertson J. (2001). Resonant Raman spectroscopy of disordered, amorphous, and diamondlike carbon. Phys. Rev. B.

[53] Stobinski L., Lesiak B., Kover L., Toth J., Biniak S., Trykowski G., Judek J. (2010). Multiwall carbon nanotubes purification and oxidation by nitric acid studied by the FTIR and electron spectroscopy methods. J. Alloys Compd..

[54] Chen J., Chen Q., Ma Q. (2012). Influence of surface functionalization via chemical oxidation on the properties of carbon nanotube. J. Colloid Interface Sci..

[55] Yu F., Ma J., Wu Y. (2011). Adsorption of toluene, ethylbenzene and m-xylene on multi-walled carbon nanotubes with different oxygen contents from aqueous solutions. J. Hazard. Mater..

[56] Motchelaho M.A.M., Xiong H., Moyo M., Jewell L.L., Coville N.J. (2011). Effect of acid treatment on the surface of multiwalled carbon nanotubes prepared from Fe–Co supported on CaCO_3_: Correlation with Fischer–Tropsch catalyst activity. J. Mol. Catal. A Chem..

[57] Kragulj M., Tričković J., Dalmacija B., Kukovecz Á., Kónya Z., Molnar J., Rončević S. (2013). Molecular interactions between organic compounds and functionally modified multiwalled carbon nanotubes. Chem. Eng. J..

[58] Davis W.M., Erickons C.L., Johnston C.T., Delfino J.J., Porter J.E. (1999). Quantitative Fourier Transform Infrared spectroscopic investigation humic substance functional group composition. Chemosphere.

[59] Wang J., Guo X. (2020). Adsorption kinetic models: Physical meanings, applications, and solving methods. J. Hazard. Mater..

[60] Tran H.N., You S.J., Hosseini-Bandegharaei A., Chao H.-P. (2017). Mistakes and inconsistencies regarding adsorption of contaminants from aqueous solutions: A critical review. Water Res..

[61] Kalhor M.M., Rafati A.A., Rafati L., Rafati A.A. (2018). Synthesis, characterization and adsorption studies of amino functionalized silica nano hollow sphere as an efficient adsorbent for removal of imidacloprid pesticide. J. Mol. Liq..

[62] Maršálek R., Švidrnoch M. (2020). The adsorption of amitriptyline and nortriptyline on activated carbon, diosmectite and titanium dioxide. Environ. Chall..

[63] Chiou C.-H., Wu C.-Y., Juang R.-S. (2008). Photocatalytic degradation of phenol and m-nitrophenol using irradiated TiO_2_ in aqueous solutions. Sep. Purif. Technol..

