# Supplementary material for: Comparative Study on the Removal Efficiency of Clomazone and Amitriptyline via Adsorption and Photocatalysis in Aqueous Media: Kinetic Models and Toxicity Assessment
Source: Materials (Basel). 2024 Mar 16;17(6):1369. doi: 10.3390/ma17061369 (PMC10972123; doi:10.3390/ma17061369)
Supplement: Supplementary file 1 [file materials-17-01369-s001.zip › materials-2866283-supplementary.pdf]

SUPPLEMENTARY MATERIAL

Comparative Study on the Removal Efficiency of Clomazone and Amitriptyline via Adsorption and Photocatalysis in Aqueous Media: Kinetic Models and Toxicity Assessment

Nataša Tot [1,†], Vesna Despotović [2,*,†], Sanja Panić [3], Branko Kordić [2], Nina Finčur [2], Jovana Prekodravac [4], Dimitar Jakimov [5], Predrag Putnik [6,*], Biljana Abramović [2], Daniela Šojić Merkulov [2]

[1] Technical College of Applied Sciences in Zrenjanin, Đorđa Stratimirovića 23, 23000 Zrenjanin, Serbia; natasazec993n@gmail.com
[2] Department of Chemistry, Biochemistry and Environmental Protection, University of Novi Sad Faculty of Sciences, Trg Dositeja Obradovića 3, 21000 Novi Sad, Serbia; branko.kordic@dh.uns.ac.rs (B.K.); nina.fincur@dh.uns.ac.rs (N.F.); biljana.abramovic@dh.uns.ac.rs (B.A.); daniela.sojic@dh.uns.ac.rs (D.Š.M.)
[3] Faculty of Technology, University of Novi Sad, Bulevar cara Lazara 1, 21000 Novi Sad, Serbia; sanjar@tf.uns.ac.rs
[4] Department of Laboratory for Radiation Chemistry and Physics "Gamma" 030, Vinča Institute of Nuclear Sciences, National Institute of the Republic of Serbia, University of Belgrade, 11080 Belgrade, Serbia; prekodravac@vin.bg.ac.rs
[5] Oncology Institute of Vojvodina, Faculty of Medicine, University of Novi Sad, Put Doktora Goldmana 4, 21204 Sremska Kamenica, Serbia; dimitar.jakimov@mf.uns.ac.rs
[6] Department of Food Technology, University North, Trg dr. Žarka Dolinara 1, 48000 Koprivnica, Croatia
* Correspondence: vesna.despotovic@dh.uns.ac.rs (V.D.); pputnik@alumni.uconn.edu (P.P.)
† These authors contributed equally to this work.

S2. Materials and Methods

S2.2. Characterization methods for MWCNTs

The structure and morphology of the prepared MWCNTs samples were characterized by TEM (FEI TECNAI G2 20X-TWIN Transmission Electron Microscope). XRD measurements were performed on a Rigaku Miniflex 600 with CuKα radiation λ = 0.15406 nm. Raman spectra of the samples were obtained using a DXR Raman Microscope equipped with a diode-pumped solid-state laser with an excitation wavelength of λ = 532 nm. The laser was coupled with a CCD camera as a detector, full range grating (900 lines/mm), 10x microscope objectives and OMNIC software for collecting and analyzing the spectra. All samples were exposed to the radiation of the laser with a power of 9 mW, six times for 30 seconds. FTIR spectroscopy was carried out on a BRUKER Vertex 70 IR spectrometer (wavenumber range of 400 – 4000 cm^{-1} with a resolution of 2 cm^{-1}) on solid samples prepared using the KBr pellet method. The quantification of basic and acidic oxygen-containing functional groups was performed using the Boehm titration method. Textural characteristics were determined by means of low-temperature N_2 adsorption/desorption method, using He as a carrier gas (Micromeritics ASAP 2010). Specific surface area was calculated by the BET equation, while mean pore diameter and pore volume were determined from the adsorption part of the N_2 isotherm and calculated by the Barrett-Joyner-Halenda (BJH) method. Pores were classified according to the Brunauer-Deming-Deming-Teller method based on hysteresis loops of adsorption-desorption isotherms.

S2.3. Measurements of photocatalytic activity

The aqueous solutions were prepared using ultrapure water. All stock solutions were protected from light and stored at room temperature. Experiments were carried out using 20 mL of emerging pollutant solution, with an initial concentration of 0.3 mmol/L for all experiments, while catalyst loading was 2.0 mg/mL for all nanoparticle types (TiO_2 Kronos and TiO_2 Aeroxide). The aqueous suspension of the catalyst was sonicated (35 kHz) in the dark for 15 min before irradiation, in order to uniformly disperse the particles of photocatalyst and to obtain adsorption equilibrium. Following the sonication process, no evidence of pollutant adsorption was detected on the catalysts surface. Irradiation under UV was performed using 125 W high-pressure mercury lamp (Philips, HPL-N, emission bands in the UV region at 304, 314, 335 and 366 nm, with maximum emission at 366 nm and intensity of 2.6×10^{-3} W/cm^2 in the visible region and 1.4×10^{-2} W/cm^2 in UV region) together with an appropriate concave mirror. On the other hand, under simulated solar irradiation was carried out using a 50 W halogen lamp (Philips) with the intensity of 0.1 W/cm^2 in the visible region and 2.2×10^{-4} W/cm^2 in the UV region. The suspension was thermostatted at 25.0 °C in the stream of O_2 (3.0 mL/min), and then irradiated. During irradiation, the suspension was stirred at a constant rate under

the continuous O_2-flow. The obtained suspensions were filtered through Millipore (Millex-GV, 0.22 μm) PVDF membrane filters. The preliminary check confirmed the absence of pollutants adsorption on the filters.

S2.5. Analytical Methods

Kinetic studies of all investigated pollutants were monitored by ultrafast liquid chromatography with diode array detector (UFLC–DAD) using Shimadzu UFLC Nexera, equipped with an Eclipse XDB-C18 column (150 mm × 4.6 mm i.d., particle size 5 μm). All chromatograms were recorded in the wavelength range from 190–300 nm. Mixtures of acetonitrile (ACN, 99.9%, Sigma-Aldrich) and water were used as mobile phase for analysis of all studied pollutants, which was acidified with mass fraction of 0.1% H_3PO_4 (85%, Sigma-Aldrich). In the case of CLO the UV/vis DAD detector was set to 210 nm (wavelength of maximum absorption of CLO) and the ACN : water ratio was 60 : 40 (v/v), following isocratic regime with flow rate of 1.0 mL/min. Also, samples of AMI were analyzed with an Inertsil ODS-4 column (2.1 mm x 50 mm *i.d.*, particle size 2 μm, 35 °C). The UV/Vis DAD detector was set at 206 nm (wavelength of AMI maximum absorption). The ratio of mobile phase for ACN and water, was 30 : 70 (v/v), following isocratic regime (flow rate was 0.4 mL/min).

Delta Ohm HD 2102.2 (Padova, Italy) was used for the UV energy fluxes measurements. The radiometer was fitted with the LP 471 UVA sensor (spectral range 315–400 nm), and in the case of visible energy radiometer was fitted with the LP 471 RAD (spectral range 400–1050 nm).

All experiments were performed at natural pH ~5 (CLO) and pH ~5.5 (AMI). The change in pH value during the degradation was monitored using a combined glass electrode (pH-Electrode SenTix 20, WTW) connected to the pH-meter (pH/Cond 340i, WTW).

Table S1. Structural parameters of the investigated photocatalysts.

Sample	Surface Area (m^2/g)	Pore size Distribution (nm)	Anatase (%)	Rutile (%)
TiO_2 Aeroxide	50.6	25.4	89	11
TiO_2 Kronos	>250.0	15.0	100	-

Table S2. Physicochemical properties of investigated pollutants.

Parameter	CLO	AMI
IUPAC name	2-[(2-chlorophenyl)methyl]-4,4-dimethyl-1,2-oxazolidin-3-one	(3-(10,11-dihydro-5H-dibenzo[a,d][7]annulen-5-ylidene)-N,N-dimethylpropan-1-amine hydrochloride
Chemical formula	$C_{12}H_{14}ClNO_2$	$C_{20}H_{24}ClN$
Molecular weight (g/mol)	239.70	313.9
Type of pollutant	Herbicide	Tricyclic antidepressant

S2.6. Cytotoxic activity

The cell line used in the study was MRC-5 (normal foetal lung fibroblasts, ATCC CCL 171). The cells were grown in Dulbecco's modified Eagle's medium (DMEM) with 4.5% of glucose, supplemented with 10% of foetal calf serum (FCS, Sigma) and antibiotics and antimycotics solution (Sigma). The cell line was cultured in flasks (25 ml, Costar) at 37 °C in the atmosphere of 100% humidity and 5% of CO_2 (Heraeus). Exponentially growing viable cells were used through the assays.

Compounds were used at concentrations ranging from 0.3×10^{-6} M to 5×10^{-6} M, to define IC_{50} concentration for every particular exposure time. The substances were added in a volume of 10 μL/well.

Growth inhibition was evaluated by tetrazolium colorimetric MTT assay (Sigma). The assay is based on the cleavage of the tetrazolium salt MTT, (3-(4,5-dimethylthiazol-2-yl)-2,5-diphenyl tetrazolium bromide), to formazan by mitochondrial dehydrogenases in viable cells. Exponentially growing cells were harvested, counted by trypan blue and seeded into 96-well plates (Costar) at optimal density of 10×10^3 cells per well to assure logarithmic growth rate throughout the assay period. Viable cells were seeded in a volume of 90 μL/well, and pre-incubated in complete medium at 37 °C for 24 h to allow cell stabilization prior to the addition of substances. Tested compounds, at tenfold the required final concentration, were added (10 μL/well) to all wells except to the control ones and microplates were incubated for 48 h. The wells containing cells without tested compounds were used as control. Three hours before the end of incubation period 10 μL of MTT solution was added to all wells. MTT was dissolved in medium at 5 mg/mL and filtered to sterilize and remove a small amount of insoluble residue present in some batches of MTT. Acid-isopropanol (100 μL of 0.04 mol/L HCl in isopropanol) was added to all wells and mixed thoroughly to dissolve the dark blue crystals. After a few minutes at room temperature, to ensure that all crystals were dissolved, the plates were read on a spectrophotometer plate reader (Multiscan Ascent, Thermo Labsystems) at 540/690 nm. The wells without cells containing complete medium and MTT only acted as blank.

Inhibition of growth was expressed as a percent of a control and the cytotoxicity was calculated according to the formula:

$$(1-A_{test}/A_{control}) \times 100.$$

The substance potency was expressed as the IC_{50} (50% inhibitory concentration).
